# How Slaughtered Animals Are Inspected at the Abattoir at Berlin, Germany

**Published:** 1884-01

**Authors:** 


How Slaughtered Animals are Inspected at the Abattior at Berlin, Germany.—The subject of meat inspection is one of great importance to the public health, yet in this eminently practical country we are far behind many European nations; in fact we have really no such thing as a well organized meat inspection at any of our slaughtering places in this country.

As a matter of instruction as well as interest, we give the following items from the official report of the Berlin Abbattior for the quarter ending in September, 1883, officials: 1; a superior veterinary inspector and ten assistants—all veterinarians: 2; one veterinarian especially appointed as "microscopic inspector:" 3; four division supervisors of the trichin-inspection department; ninety sub-inspectors; thirty collectors of specimens; four persons to brand the inspected articles; total, 141 persons.

*At the Boston Abattior there is one inspector.*

From July 1 to September 30, 1883, there were slaughtered 22,913 cattle; 19,461 calves; 47,156 sheep; 52,430 swine.

---

Condemned, before slaughtering; 30 cattle, 2 calves, 4 sheep, 357 hogs.

Condemned organs; from cattle, 5,768; calves 40; sheep 1,530; swine 1,565.

710 cattle; 2 calves; 465 swine were condemned as unfit for food on account of tuberculosis.

Tubercle-bacteria were found in all newly developed tubercles, but failed in those which had undergone fatty metomorphosis or calcification.

252 hogs were condemned on account of "measels;" 56 swine on account of trichiniæ.

Ecchinococci were found in the *lungs* of 1,145 cattle; 700 sheep; 224 swine; and in the livers of 273 cattle, 415 sheep and 322 hogs.

Filaria in the lungs of 13 sheep and 179 swine. B.

